# Teachers’ Ideological Dilemmas During the Pandemic at Higher Education Institutions: a Discursive Psychological Approach

**DOI:** 10.1007/s42087-022-00292-9

**Published:** 2022-06-20

**Authors:** Kyoko Murakami, Sachihiko Kondo, Jianzhong Hong

**Affiliations:** 1 University of Bath, Bath, UK; 2 Osaka University, Osaka, Japan; 3 Central China Normal University, Wuhan, China

**Keywords:** Online teaching, The pandemic, HE teachers, Ideological dilemmas, Common sense

## Abstract

The COVID-19 pandemic has impacted higher education (hereinafter “HE”) teaching and learning approaches globally since 2020. It has compelled a major shift from face-to-face to online delivery, affecting the ways HE teachers teach and communicate with students. In this paper, we explore an under-researched area, teachers’ ideological dilemmas surrounding online teaching and issues related to remote or technologically mediated communication adopted in three countries, China, Japan and the UK. Drawing on the theoretical and methodological framework of discursive psychology, we focus on the concepts called ideological dilemmas and the kaleidoscope of common sense in order to examine common sense views of HE teachers regarding online and onsite teaching as well as blended learning where these constitute ideological dilemmas. Taking an exploratory, small case study approach, we present a discourse analysis of interviews with HE teachers and reveal their ideological dilemmas over online teaching. We identify the patterns of shifting justifications in the interviews. Our discussion highlights the dynamic and dilemmatic nature of the HE teachers’ views, some of which are shaped by the local university context, as well as the different ways in which the spread of COVID-19 is taking place and the various measures taken by each country’s government against the damaging effects of the pandemic.

## Introduction

The COVID-19 pandemic has impacted higher education (hereinafter “HE”) teaching and learning approaches globally since the spring of 2020. It has compelled a major shift from face-to-face to online delivery, affecting the ways in which HE teachers teach students, support their learning, and communicate with them. Lockdowns have compelled them to engage with online teaching as a default mode, having now worked from home for many months. Thanks to digital technologies, online teaching has become the main mode of delivery and implementation in institutions and teaching. Teaching and learning at HE institutions have continued, albeit with some disruptions in some countries. Since online platforms and delivery have become the default mode for HE teaching and learning,^1^ there has been an apparent trend within social science research to focus on topics such as students’ attitudes toward online and remote communication with their teachers (Mpungose Cedric, 2020), student mental health issues such as anxieties caused by being in lockdown and frustrations over online teaching and learning, as well as broader institutional or national education policy research (Mishra et al., 2020). In contrast, only a few studies on HE teachers have been identified, on topics including their experience of webinars (Cleland et al., 2020), teachers’ dissatisfaction with distance education in Turkey (Durak, 2020), HE teachers’ technostress in Spain (Penado Abilleira et al., 2021), and wellbeing in dealing with isolation in England (Kotera, 2020).

Although these studies examine the experiences of teaching and teacher wellbeing, the theoretical frameworks used to investigate the impact of COVID-19 do not produce nuanced accounts of how the experiences, attitudes, and opinions of HE teachers are shaped within lived ideologies in a given culture. In this paper, we would like to shed light on this under-explored area of teachers’ ideological dilemmas surrounding ‘pandemic pedagogy’ (Gurung, 2020), including online teaching and issues related to emergency remote or technologically mediated teaching and pedagogic communication adopted in three countries, China, Japan, and the UK. Pandemic pedagogy is a phenomenon that is still evolving in these particular settings. This is where the variability of opinions, views, and attitudes among HE teachers are seen as ideological dilemmas (Billig et al., 1988). We address the following research questions. What ideologies and sense-making practices are present in HE teachers’ talk about teaching through the pandemic? How do local institutional guidelines implemented during the pandemic intersect with teachers’ thinking about HE teaching and their role as an HE teacher?

In the following section, we first present the background and context that are relevant to our discourse analysis of HE teachers’ dilemmas over online and face-to-face teaching. We then provide a discussion of our theoretical framework, drawing on ideological dilemmas (Billig et al., 1988) and the kaleidoscope of common sense (Billig, 1992). This underpins our work on examining the common sense views of HE teachers about online and onsite teaching as well as blended learning, which constitute ideological dilemmas, and identifying the patterns of shifting justifications. Our research takes an exploratory, small case study approach, presenting a discourse analysis of interviews with HE teachers and revealing their ideological dilemmas surrounding online teaching. We discuss the dynamic nature of teachers’ ideological dilemmas some of which are shaped by the local university context, as well as by the different ways in which the spread of COVID-19 is taking place and the various measures taken by each country’s government against the damaging effects of the pandemic.

### Background and Context

We situate our discursive analysis in the relevant context, highlighting the commonalities and differences between the three countries which we discovered in recent research on HE teachers’ views, satisfaction, and concerns, focusing on a speedy, radical shift from face-to-face teaching^2^ to an online teaching–learning^3^ platform during the pandemic. This shift was not optional. It is important to recognise that the discourse of online teaching by HE teachers in these three contexts is not a discourse on how they chose online teaching and why they were not asked to make an ethical choice between online and face-to-face teaching (see Weinberg, 2014). The HE teachers’ discourse is based around online teaching being enforced as an inevitable consequence of the pandemic.

### From Face-to-Face to Online Teaching During Lockdowns

The lockdown of Wuhan on January 23, 2020, surprised China and the world. To prevent the further spread of COVID-19, colleges and universities in China were instructed to carry out online teaching actively, ensuring that the national initiative of ‘Suspending Classes Without Stopping Learning’ be implemented efficiently (Ministry of Education of the People's Republic of China, 2020; Zhang et al., 2020). Under these circumstances, all universities were forced to deliver teaching online from March 1, 2020. A shift to online teaching was thus implemented, although the process was a little slower than expected. For online teaching, professors were fully responsible for all courses they were to deliver. They could, for instance, decide which technical platforms (e.g. Zoom, Tencent meeting) and lecture formats (e.g. live lectures, recorded lectures) to use. The quality of online teaching was entirely in the teachers’ hands. They were also allowed to decide if they would hold the final examination online, or rather wait for a regular face-to-face examination when all students were permitted to return to campus. As the pandemic gradually came under control in China, students from Wuhan were allowed to return to campus, in stages, from June 2020. All universities in Wuhan reopened between late August and early October 2020, when the Fall Semester started. Currently, at the time of writing, all universities have returned back to normal teaching, with strictly controlled access to campus. However, new international students enrolled in 2020 are still taking courses online.

In responding to the UK Prime Minister’s statement on the coronavirus on 12 March 2020, UK universities advised teachers to be prepared to move to online delivery, recording lectures and updating VLE both for students who were self-isolating and for those needing to come to campus to receive teaching. Many academic staff had little or no experience with online teaching (Ma et al., 2021). Universities provided staff resources and information on ensuring as much coverage across the board as possible, whilst developing contingency arrangements for emergency remote teaching. On March 23, 2020, the PM announced the first lockdown in the UK. Some of the measures observed across the university sector included shifting to online delivery of teaching and learning, encouraging home working by students and staff, postponing March/April graduation ceremonies, and changing examination arrangements. In June 2020, upon the easing of lockdown restrictions, universities reviewed their teaching, learning, and assessment to ensure that the required flexibility to deliver a high-quality experience and support for students’ learning and achievement was in place. Many universities published plans for the September term starting in 2020. They planned to provide courses through blended learning, which refers to a course that includes both online and face-to-face elements. Universities UK reported that despite universities’ effort to ensure that teaching continued online, nothing could replicate the benefits of face-to-face interaction, and many students were said to have been overwhelmed by high levels of screen time (2021).

On April 7, 2020, the Japanese government announced a state of emergency for major cities such as Tokyo and Osaka to prevent COVID-19 and extended the announcement nationwide on April 16, closing schools at the beginning of the Japanese academic year. According to a May 20 survey by the Ministry of Education, Culture, Sports, Science, and Technology (hereinafter “MEXT, 2020a, b”), only 10% of the HE institutions in Japan were conducting face-to-face and hybrid-style teaching, with 90% of them offering online lectures only. States of emergency were announced intermittently in accordance with the level of pandemic. During the first state of emergency, most universities sustained campus closures, but gradually allowed their students to join classroom lectures with measures in place to prevent infection. In July 2021, it was reported that 97.5% of the universities offered exclusively classroom and hybrid lectures during the spring semester of 2021 (MEXT, 2021). The HE institutions which offered virtual lectures exclusively, or for more than 30% of classes, were as few as 28 schools out of 1064 institutions in Japan.

### Hardships and Challenges for Teachers

The Central Committee of the Faculty and Staff Union of Japanese Universities issued “A Report into Labour, Education and Research Conditions During the Novel Coronavirus Pandemic” in October 2020, based on a nationwide survey of union members. The majority of academics surveyed felt that their workload had become heavier in mid-2020; 47.5% of the respondents claimed that they were forced to work a considerable amount of overtime, and 32.9% said that they were experiencing a somewhat greater workload than in previous years. There were several reasons for this overtime, such as setting up new teleworking infrastructures at home (at their own expense) and the additional efforts related to infection prevention, on top of regular teaching and research routines. One of the burdens stretching academic staff was preparation for and implementation of the newly introduced online teaching. Professors and lecturers were forced to redesign their lectures, from scratch, in 2020. They needed to convert their in-person lectures into on-demand, hybrid, or other appropriate online teaching formats. On top of this, they had to also prepare extra follow-up sessions and new forms of assessment appropriate for online learning. HE teachers in China faced similar challenges (Wu & Li., 2020). Some teachers, especially senior teachers, had no online teaching experience, let alone of courses taught online only. Training for online teaching was provided in different ways, and some teachers proactively joined training sessions, but most could only try their best. Similarly, UK teachers experienced an increased workload and required extra time to get upskilled in new technologies to meet the urgent demands of online teaching (e.g. Bachmann, 2021; Dulohery et al., 2021; Jasi, 2021; Ma et al., 2021).

### Higher Education: Teaching, Learning, or Something Else?

HE institutions have diversified functions. They are expected to act as both research and educational institutions simultaneously. With respect to the latter function, Billig and others suggested that ‘the very term “education” is a symbol of combining contradictory themes’ (1988: 60). The word comes from the Latin *e-ducare,* which means to ‘lead out’, as many modern progressive teachers are aware. Ever since the ancient past, it has been broadly believed that good teachers are facilitators of ‘discovery learning’. On the contrary, one may consider the *in-duction* style or knowledge-transmission teaching as conservative, old fashioned, and anachronistic. Whatever the best approaches toward education may be, the art of teaching relies on teachers’ individual skills.

In the following empirical sections, we will outline our theoretical and methodological framework, inspired by discursive psychology, and its approach to examining the HE teacher discourse in terms of ideological dilemmas. We will analyse interviews with teachers focused on ideological dilemmas concerning, for example, teaching and learning, different generations and flexibility, institutional and individual beliefs, and the advantages and disadvantages of technology-oriented education.

## Theoretical and Methodological Frameworks

### Discursive Psychology and Social Construction

Our work featured in this article is methodologically situated within discursive psychology, underpinned by a social constructionist paradigm (Potter & Wetherell, 1987). Discursive psychology takes an approach to everyday discourse, treating talk and text as part of social practices instead of as a reflection of inner cognitive processes (Wiggins, 2017). Taking a relativist stance, discourses are argued to be both constructed by and constructive of the world. At the macro-level, discourses are often used for everyday sense-making of institutional realities (Garfinkel, 1967/1984); at the micro-level, local adaptations and orientations of discourse can be observed in various situated functions, such as justifying the speaker’s own opinions, accountability, or social status (Gill, 1993; Wetherell & Potter, 1992). Discursive psychology provides a framework to consider how culturally available understandings provide the context for sense-making in local interactions.

### Rhetoric, Ideological Dilemma, and the Kaleidoscope of Common Sense

One of the leading discursive psychologists, Potter (1996) defines discourse as ‘talk and texts as parts of *social practices*’ (p. 105; emphasis original). Potter encourages analysts to look at the micro-details of ongoing speech and texts (see Burr, 1995, p. 184 for different definitions of ‘discourse’), and this school of constructionists often refers to the less dynamic picture of ‘discourse’ (e.g. ‘system of statements’ by Parker (1992) or the Foucauldian idea of discourse) as either ‘commonplace’ or ‘interpretative repertoire’. Commonplaces are viewed as ‘representing values which themselves are not matters for debate but which rhetorically are often used to support contestable positions’ (Billig, 1991 p. 208). The term ‘interpretative repertorie’ is defined as ‘a lexicon or register of terms and metaphors drawn upon to characterise and evaluate actions and events’ (Potter et al., 1987, p. 138).

Among discursive psychologists, who are critical of a traditional social psychological conceptualisation of attitude, Billig (1989) examined rhetorical organisations in everyday conversations, through which speakers variably represent themselves as theoretically inconsistent but practically consistent. Even a person who claims to have a ‘strong view’ continuously negotiates the contents of his/her view—sometimes in contradictory ways at different times; nevertheless, he/she retains the status of ‘the holder of a strong view’ (Billig, 1989, 1992). Billig et al. (1988) reveal the flexible and extensive nature of rhetorical formulations in everyday sense-making. Billig names these phenomena the ‘kaleidoscope of common sense’ (1992), whereas some theme-dependent rhetorical organisations can be named separately (e.g. ‘royal credit’ discourse [1992]).

Analysis of discourse, especially of its rhetorical formulations, could identify commonplaces and interpretative repertoires, which people use as resources to support their own views (Potter, 2012). Being critical of the ways in which cognitive psychology and social theory approach common sense reasoning, Billig et al. (1988) claim that a social reality is normatively organised through contrary themes and is essentially argumentative. Based on this understanding, Wetherell and Potter analysed Pākehā New Zealanders’ discourse on land, language, and affirmative action, revealing ‘a particular set of ideological dilemmas played out in the commonplaces of political argument’ (1987, 176). They explored how the various formulations of these dilemmas led to the maintenance of racist practices as a ‘bricolage in action as people draw on contradictory resources in a flexible and variable fashion to construct their accounts’ (p. 176). As another example of this kind of research of rhetorical formulations, Kondo looked at the modern constitutional monarchy in Japan (2000). A variety of naming practices and levels of politeness allowed Japanese speakers to describe the emperors and imperial family as only ordinary mortals, but as distinguished superiors with inherited social status. They frequently shifted between interpretative repertoires of formality and informality, in ways which resembled the phenomenon of ‘kaleidoscope of common sense’ (Billig, 1992, p. 149).

Ideological dilemmas in discourse enable us to examine HE teachers’ utterances as parts of local negotiations, demonstrating where they position themselves regarding HE teaching in the pandemic. Throughout our interview analysis, the teachers’ discourse is concerned with today’s tertiary education, such as the different levels of technological accommodation and the degree of positivity towards digitalised teaching. We focused on this theme, online teaching, which was attributed differently in the interviews in accordance with the degree and scale of institutional initiatives, digital literacy skill levels of individual staff, personalities, generational issues, and cultures of the HE institutions.

### Research Design

The present article has evolved from a series of exploratory discussions among the authors regarding the changes in our own workplaces, universities, and national policy and strategies combating COVID-19 in China, Japan, and the UK. This study adopts a small-case study design (Yin, 2009), with the case being the phenomenon of ideological dilemmas of the HE teacher research participants in the three universities in China, Japan, and the UK.

### Data Collection Method^4^

A total of eight HE teachers^5^ were selected for one-to-one, open-ended interviews using opportunity sampling (Kvale & Brinkmann, 2009). The selection of the participants was not pre-set, but rather was ‘conceptually driven’ by discursive psychology, the theoretical framework underpinning our research (Silverman, 2010, p. 23). The interview schedule was developed in accordance with the research questions, along with a standardised interview question: ‘Are you happy about online teaching?’ The interview question was designed to elicit responses from the participants. Interviews were conducted between mid-December 2020 and February 2021 by the authors, in our respective universities, and in our own working languages. Due to social distancing policy in our universities, we used Zoom (or a video conference software equivalent to Zoom) to interview participants. We used Zoom’s video (and some audio) recording function to generate video and audio files and transcribed the interviews. Once rough transcripts were made, initial analytical memos were incorporated into the transcripts, followed by a finer transcription in verbatim using the abridged version of Jeffersonian transcription notations (Atkinson & Heritage, 1984).

## Research Ethics

The current project is an exploratory study, arising out of our conversations. We hope to develop a larger study for international research collaboration. Oral permission was obtained from the interview participants by the interviewers prior to all the interviews, and consent was audio-recorded. We each followed our respective university’s research ethics guidelines, especially ensuring informed consent, anonymity, and confidentiality, along with matters relating to General Data Protection Regulation (GDPR).

## Analysis

The analytic process began by coding the interview transcripts to identify patterns in meaning-making that were organised around recurring themes and commonplaces toward online teaching. After several rounds of re-reading the transcripts, the central ideological dilemmas were identified as to how the HE teachers position themselves around the mandatory online teaching imposed by the university or the government. Various commonplaces, interpretative repertoires, and HE teachers’ beliefs about online teaching were mobilised to justify their answers to the interview questions. The participants’ ideological dilemmas in the ongoing talk were examined by focusing on the processes of construction of various meanings associated with the HE teachers’ views on online teaching and particular social actions being performed (Potter & Wetherell, 1987). In other words, the focus at this latter stage of the analysis was on what these constructions have accomplished (see, e.g. Edley, 2001).

### Capable Junior Colleague Becomes Happy and Energetic

Some background information is necessary for Extracts 1 and 2. In October and November 2020, Japan temporarily eased its border controls for valid visa holders, including non-Japanese students. Although this policy was revised immediately after the pandemic became serious again, at the time of our interviews, a small number of newly enrolled international students had successfully entered Japan and were studying in their dormitory rooms under strict travel restrictions. In the following extracts, a young social science associate professor (Y), with four years of teaching experience, started her account haltingly. Interviewer S has almost twenty years of experience at the same university. Y and S, who are both Japanese, received their advanced degrees from US and UK universities, respectively, and this interview was conducted in English. The initial question posed by S, which was the same as in the other interviews, was ‘Are you happy about teaching online?’ She firstly replied, ‘I have different feeling in the period of pandemic’. Responding to this statement, interviewer S pressed her on the question in lines 1 and 2.

**Figure 1:**
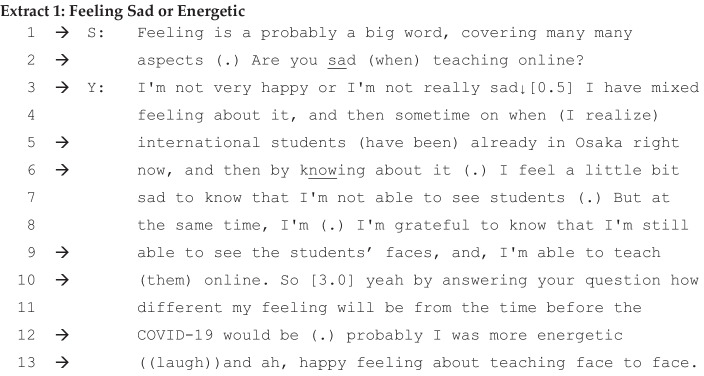

Interviewer S requests a junior staff member of the same university to clarify the detailed meaning of her ‘feeling’ (lines 1–2). The term ‘feeling’ itself implies little. However, together with some adjectives, it may refer to a variety of spectrums, such as good-bad, positive–negative, joyful-sad, and others. Interviewer S tries not only to neutrally clarify the details of her feeling, but also to invite her agreement (Pomerantz, 1984) with the assessment criteria ‘sad’: ‘Are you sad (when) teaching online?’.

The antonyms of ‘happy’ could be ‘depressed’, ‘troubled’, ‘dissatisfied’, or simply ‘unhappy’, but Interviewer S uses ‘sad’, and their discussion develops along the trajectory of the ‘happy versus sad’ spectrum. The choice of English antonyms by a non-native English speaker is not the focus of this paper. We, instead, witness how the dilemmatic nature of the teaching platform could be constructed along the lines of ‘happy-sad’ and other spectrums, which are part of the interpretative repertoires of ‘pandemic and online teaching’ discourse. It is immediately recognisable that the senior professor, S, invites the junior colleague, Y, into repertoires within the spectrum close to ‘sad’. He does not position himself as happy about online teaching (Harré & van Langenhove, 1998) and expects Y, his junior colleague, to agree with his view. At first, she consents to describing her feeling within the ‘happy-sad’ spectrum, but moderates it by adding that her feeling is not an extreme one, neither very happy nor really sad. After a long silence (0.5 s), she goes on to elaborate on her ‘mixed feeling’ (line 3 onwards).

What is important to highlight here is that, in the next sequence, she clarifies that her sadness comes from the fact that she is forced to teach international students online, even though these students are at an accessible distance (i.e. Osaka). Her sadness comes from ‘knowing about’ this constraint, which makes her position dilemmatic; but, unlike her senior colleague, she does not position herself as someone who is sad or powerless because of online teaching obligations (Davies & Harré, 1990; Harré & van Langenhove, 1998). In line 6, a kaleidoscope of common sense is turned as she elaborates on her feelings (Billig, 1992): Even with the online teaching method, she could, at least, see or view students’ faces on screen. From her point of view, seeing students’ facial impressions offers her a similar teaching experience, just using technology.

In the following formulation, she firstly cancels her previous responses. She explicitly comments that she has not answered the question properly, but that she will do so in the following account, interposing a signalling phrase, ‘by answering your question’. Schegloff et al. (1977) claimed that corrections of utterances are often placed to avoid conflicts. However, here in line 10, the junior professor repairs her previous formulation; then, next she challenges her senior, who positions himself as dissatisfied with online teaching requirements; she presents new interpretative repertoires or commonplaces from line 12 onwards. Despite Asian values which hold for senior-junior relationships, the young associate professor does not accommodate her own role or footing (Goffman, 1981) in relation to her senior. The senior proposes an ‘online teaching discourse’ within the ‘happy-sad’ spectrum, with particular emphasis on the ‘sad’ dimension. Revising this, the young professor emphasises the ‘happy and energetic’ dimension.

In Extract 2, which immediately follows Extract 1, Y, the junior academic, turns the ‘kaleidoscope’ further and introduces novel spectrums of ‘learning-teaching’ and ‘capable-incapable’ as she accounts for her own online teaching experience.

**Figure 2:**
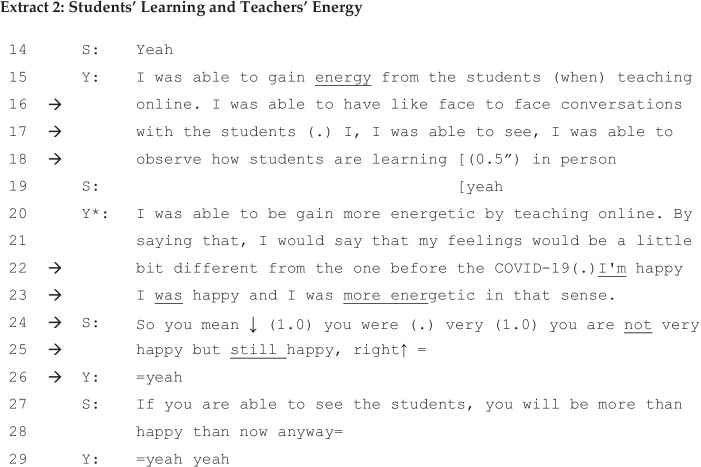

Between lines 15 to 18, she shifts the focus from lecturers to learners. The associate professor becomes energetic because she recognises her own capability, which she had not previously realised. Thanks to technology, she was able to see and observe her students and check their progress, and in lines 22 and 23, because of this, she is not only happy but energetic. She positively redefines digitised education; although the senior professor proposed it as an input-oriented burden on teachers, the junior reconstructs it as a positive and effective education framework, an output-oriented student-centred learning platform.

Her accounts are unexpected for the senior professor, in opposition to his own. In order to mitigate potential conflicts between the two, the senior (S) tries to rephrase her account, ignoring her ‘happy and energetic’ version. He goes back to the ‘happy-sad’ spectrum or, borrowing Billig’s metaphor further, turns the kaleidoscope backward, in lines 24 and 25. She does not argue back against his interpretations and again, he oppresses her by his interpretations. In these interactions, the senior demonstrates his influence upon the account as a whole and invites the agreement of the younger interviewee in question form. In order to avoid making her senior lose face (Brown & Levinson, 1987; Goffman, 1955; Scollon et al., 2012), the junior avoids further disagreement or conflicts and replies with a short ‘yeah’, demonstrating that she has stopped resisting her senior any further. Throughout Extracts 1 and 2, discursive psychology reveals that the kaleidoscope of common sense (Billig, 1988) of university online teaching is turned forward by the younger and backward by the elder generation, with reference to the repertoires of the ‘happy-sad’, ‘capable-incapable’, and ‘teaching–learning’ spectrums, in accordance with the positions held by the speakers in the university teaching environment during the pandemic period.

### Interviews with Teachers in a Chinese University: Online Versus Face-to-Face and Future Versus Tradition

In this section, firstly, we explore the formulations of ideological dilemmas by teachers in University C, a university in China. During the early phase of COVID-19, the emergency policy initiative ‘Suspending Classes Without Stopping Learning’ was implemented by the Chinese government, as introduced in the section, Hardships and Challenges for Teachers. However, recent research on this policy points out that ‘there is ambiguity and disagreement about what to teach, how to teach, the workload of teachers and students, the teaching environment, and the implications for education equity’ (Zhang et al., 2020, 1). From a discursive psychological perspective, the policy worked like a slogan, as it invited a variety of commonplaces and interpretative repertoires that would resource teachers’ ideological dilemmas. Professors understood that online teaching was a necessary measure under the given circumstances, and the Chinese educational authorities assumed that the quality of education should remain at the same level, although the teaching platform had shifted. When Interviewer R asked the interviewees for their views about the slogan, however, they expressed somewhat different concerns, while all seeming to agree that it could never be more than partially realised for several reasons. P01, a professor, who has been teaching educational psychology at University C since 2005, comments on the slogan as follows:

**Figure 3:**
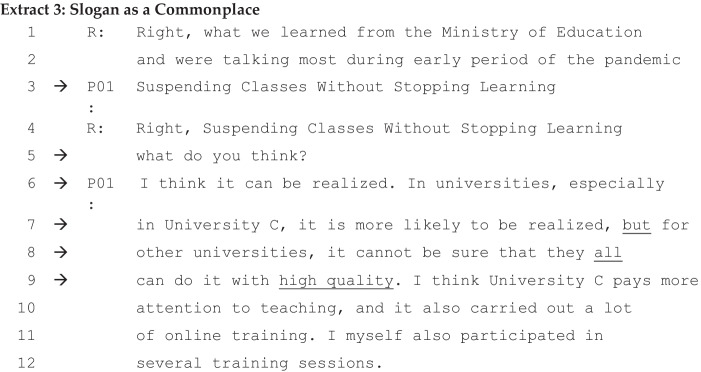

Teacher P01 seems to display a rather positive attitude toward the policy, with little scepticism. This, of course, also reflects to some extent the time at which the interviews were conducted. The conditions of HE institutions had already reverted to nearly normal, as all Chinese teachers and students had returned to campus. We also need to bear in mind that University C, where P01 works, plays a leading role in its application of educational information technology, and it has developed more than five thousand courses on its online teaching platform, Cloud Classroom, which it also developed in-house. Almost all teachers and students had some experience of online teaching before the pandemic.

For discursive psychology, the policy as a slogan itself calls for ideological dilemmas, precisely due to the variability of people’s interpretations when it comes to implementing the slogan, which is ambitious, and has already called for dissenting voices (Zhang et al., 2020). When P01’s view on the slogan was asked for, her formulation of ‘can be realised’ (l. 6) is noteworthy. By implication, the slogan is difficult to implement, but it can be done because her university is a leading figure in educational information technology and can provide high-quality online teaching. Her claim that University C can realise the policy is further warranted by her comment that she herself has taken part in online teaching training sessions. Her account is resourced with extreme case formulations, which work to justify her claim that her university, unlike other universities, can realise the policy (lines 8: ‘all’, 9: ‘high quality’, 10: ‘a lot’) (Pomerantz, 1986).

The government slogan ‘Suspending Classes Without Stopping Learning’ is an established and embedded interpretative repertoire, which is well shared amongst the interview participants. They talked about Cloud Classroom as evidence of their advantage. Some spectrums of the ‘online-face-to-face’ and ‘capability-incapability’ of delivering digital education are represented. The teachers interviewed in University C also addressed the issue of different forms of knowledge, such as acquiring declarative or procedural knowledge (Anderson, 1982; Hong et al., 2018; Schunk, 1986). In the following extract, two teachers (P02 and P03), interviewed on different occasions, shared the same view that online teaching is good for digitised learning, but not for all types. On the one hand, online teaching is suitable if the teaching content is more concerned with the acquisition of declarative knowledge; on the other hand, face-to-face is a good method if it deals mainly with the acquisition of procedural knowledge.

The following, Extract 4, reveals P02’s view, which is formulated around commonplaces in contrary terms—traditional teaching versus future-oriented online teaching. Notice how P02 categorises online teaching as combined with future-oriented and contrasted with traditional teaching.

**Figure 4:**
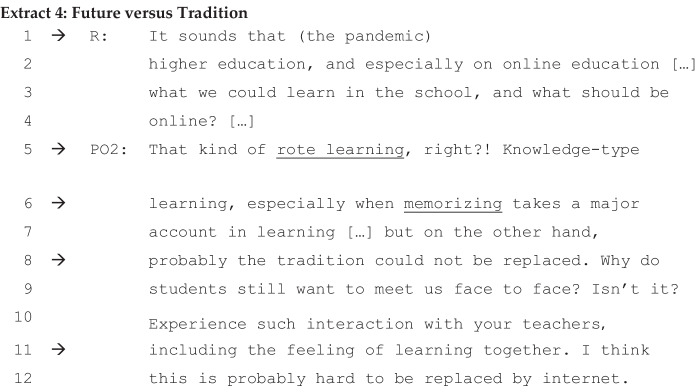

There seems to be a commonplace that the application of teaching approaches should take place depending on the teaching content. Different interpretative repertoires used to impact on future higher education (line 1) and knowledge-type learning (line 5) (i.e. traditional-style teaching) are contrasted. However, it is claimed that tradition could not be replaced (line 8) and this remains an important spectrum for the nature of education. In line 11, the ‘feeling of learning together’ can be considered as an interpretative repertoire which emphasises the common sensory advantage of face-to-face teaching. According to her personal opinion, real physical interaction between teachers and students has an irreplaceable advantage, and this is commonly understood by those who have been fully adapted to a traditional teaching style.

P03, a lecturer, seems to echo this spectrum of future versus tradition in Extract 5 below. Interestingly, in the following extract, the personalised opinion of P02 is expressed as a collective view by another interviewee, P03, in Extract 6.

**Figure 5:**
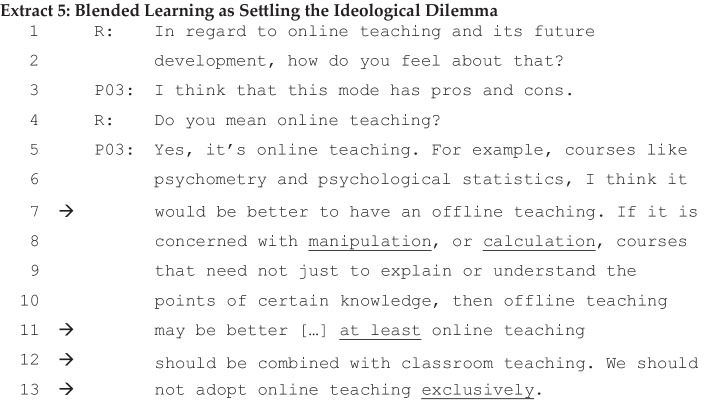

In this extract, P03, a lecturer in educational psychology, answers initially that online teaching has pros and cons. When asked to elaborate, P03 reveals her ideological dilemma, as if a kaleidoscope of common sense begins to turn. The entire sequence has a persuasive orientation (ll. 5–13). Her elaboration of ‘this [online] mode has pros and cons’ is formulated within a complex linguistic structure of conditionals and contrasts (if X, then offline teaching, but if Y then online combined with classroom teaching (ll. 7–12)). The spectrum of online-offline teaching works as a discursive resource to justify her view (l. 13) and settle her ideological dilemma. In answering the question again in reformulation (12–13), a collective pronoun of ‘we’ is used instead of the first person ‘I’. According to Mühlhäusler et al. (1990), the pronoun ‘we’ is often used not only in exclusive reference to the speaker, but also even in reference to a group that does not include the speaker. It is possible that the pronoun ‘we’ indexes a collective imagined community voice of HE. It is also possible that it is the general function of the utterance to disambiguate the meaning, removing uncertainty from her initially ideologically dilemmatic view.

### Ideological Dilemmas of a Teacher in a British University

In this section, in applying the kaleidoscope of common sense further, we explore the discursive formulation of ideological dilemmas by an HE teacher, T, in a British university. How are these accounts of online teaching used to support T’s own construction of events such as the changes of mode of teaching delivery and other important concerns for HE teachers? We shall address this analytical question by focusing on online and face-to-face teaching as the participants’ categories (Edwards & Stokoe, 2004): in this case, Interviewee T’s categories. They are designed to be used in talk, and they represent particular ways of ordering the world in which his teaching practice is based (Wetherell et al., 2001, p. 168). They are shaped in the binary contrast structure, e.g. online versus face-to-face, as a powerful, general-purpose discursive device for constructing the world as such. That makes them ideally suited to ideological, dilemmatic, and rhetorical discourse (Billig et al., 1988) and to the mundane, situated production of contrasts and alternatives (Edwards, 1997, p. 237).

The interpretative repertoires entail separate ways of talking about or constructing a participant’s category, online teaching (Gilbert & Mulkay, 1984; Wetherell et al., 2001), as they are part and parcel of the HE community’s common sense, providing a basis for the kaleidoscope of common sense, which is seen in the patterns of shifting justification (Billig, 1992).

Extract 6 below is taken from the interview with T, who constructs online and face-to-face teaching as being separate, with face-to-face taking primacy over online teaching.

**Figure 6:**
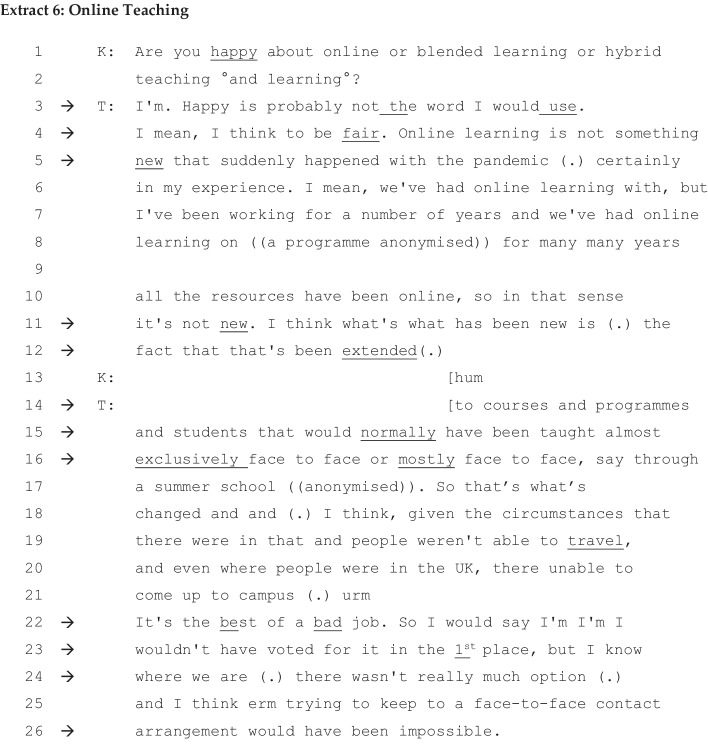

There are two interpretative repertoires of online and face-to-face teaching in Extracts 1 and 2. The first interpretative repertoire is a notion of online teaching as being not new (‘online learning is not something new’ in lines 4–5, ‘not suddenly happened with the pandemic’ in line 11). T supports this claim: He goes on to describe one of the programmes as an example of online teaching that he and his colleagues have been doing for a long time before the pandemic. It has been practiced on distance learning-based programmes in the department, and he himself has been involved in online teaching (lines 6–10). By implication, this leads to his claim of capability, that he, along with his colleagues, is well experienced, skilled at, and capable of online teaching. This is marked by extreme case formulation (e.g. ‘for a number of years’ in line 7, ‘for many, many years’ in line 8, ‘completely at a distance’ in line 9, ‘all the, all the resources have been online’ in lines 9–10) used as a way of legitimising his claims (Pomerantz, 1986).

The second interpretative repertoire is that online teaching is something new (lines 11–12). Online teaching has been ‘extended to…programmes that are normally face-to-face’ since the pandemic (lines 12 and 14–17). The interpretative repertoire of online teaching as new is presented as an impact of the pandemic. These two Interpretative repertoires in T’s account echo what seems to be a dilemma for many HE teachers. It is the dilemma of having to deliver online teaching on the programmes normally run as face-to-face for students (see also Extracts 1 and 2). The dilemma is due to new COVID-19 teaching and learning strategies from the university and its social distancing and other health and safety measures. His dilemma is settled in mitigated disagreement as ‘the best of a bad job’ (line 22) and ‘I wouldn’t have voted for it’ (line 23). He glosses over his stance by saying that there is ‘not much option’ (line 24), showing his full awareness of the situation, something that marks his experience (lines 23–24). The pandemic situation makes it impossible for T (and all HE teachers) to teach students who live on campus or commute locally face-to-face (line 26).

Implicit within his argument is an assumption that HE teachers, such as T, are competent to/capable of delivering online teaching as well as face-to-face, a view that we see being made much more explicit in the following extract. It is within this composed disposition of an HE teacher using online teaching in the pandemic that his capability claim is sustained as credible.

**Figure 7:**
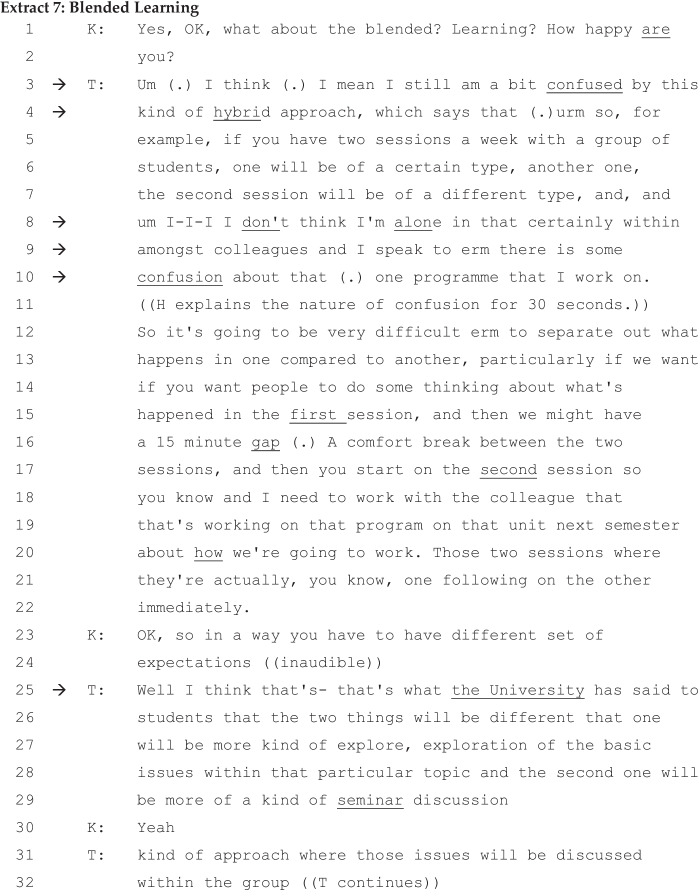

In this extract, we can see two different versions of blended learning presented in opposition. In answering K’s question on blended learning, T distances himself from the category blended learning (‘I still am a bit confused by this kind *of* hybrid approach’ [lines 3–4]), furnishing a mitigated complaint. After displaying his knowledge of how a hybrid approach ought to be implemented, he then goes on to justify the complaint based on his recent episode, normalising and authenticating the confusion (‘I don’t think I am alone in that…there is some confusion about hybrid approach’ [lines 8–10]).

Having distanced himself from blended learning as the university’s category (‘confused by this kind of hybrid approach’ [lines 3–4]), T does not attempt to overtly deny the importance of online teaching and the blended approach. However, he expresses his dissatisfaction with blended learning due to the way in which the university communicated its decision (lines 25–26). Blended learning is constituted as not being part of T’s category. The university’s blended learning approach (lines 13–20) puts two sessions one after another, without a substantial break between the two sessions, making it difficult for students to digest and reflect on the content (lines 13–20). Two interpretative repertoires of blended learning are available here: The university’s approach and the way HE teachers like T have been putting it into practice. Turning the kaleidoscope of common sense on these interpretative repertoires of blended learning creates a dilemma for T. The university’s blended learning does not work in practice in T’s view. What is more, the implementation of the university’s form of blended learning does not lead to real thinking (lines 13–22). Following T’s claim of capability for both online and face-to-face teaching, the university’s blended learning is problematised and expressed in dissatisfaction and as a mitigated complaint.

The nature of T’s ‘confusion’ is constructed not around his incompetence, inexperience, or incapability of delivering a hybrid approach including blended learning. On the contrary, his claim of capability is at the forefront of what he says. The ‘confusion’ is attributed to the university’s version of blended learning. By implication, blended learning as implemented by the university is blameworthy, as he describes how poorly the university’s blended learning panned out in practice (‘Those two sessions where they’re actually…one following on the other immediately’ [lines 20–21]) and created a dilemma for a capable teacher such as T. To make this claim even stronger, in lines 25–29, T points out the way in which the university goes about communicating this very important teaching strategy without consulting the teachers (lines 25–29). The delicate nature of complaining about the university’s blended learning is the source of T’s dilemma.

## Discussion

### Generational Discourse in Japan

The interaction between the senior male professor and the young female associate professor at University J reflects the hierarchical, cultural nature of the interactional setting. A senior-junior relationship is strictly observed in Japan, and interactions are sustained by mitigating face-threatening acts for seniors (Brown & Levinson, 1987) or demonstrating socially appropriate discernment (*wakimae*) by juniors (Ide et al., 1992). In terms of research methodology, this interaction might be an instance of “power asymmetry in qualitative research interviews” (Kvale et al., 2009, p. 33), possibly with gender-related power asymmetry. This is a specific professional conversation on teaching, not ‘a completely open and free dialogue between egalitarian partners’ (Kvale et al., 2009, p. 33). The example of Y has made visible the process of Y’s ideological dilemma being co-constructed with S with contrasting spectrums. We recognise this unique Japanese context, where ideological dilemmas about online teaching are made visible. Various spectrums (e.g. teaching–learning, capable-incapable) emerged, and they were reformulated co-constructively within the course of the interaction.

Secondly, what we have witnessed in Extracts 1 and 2 is that social identities were in the making, in which the junior professor, being interviewed by the senior professor, explored her view on mandatory online teaching due to the pandemic. Her interactional positions were oscillating moment-by-moment (Cook, 2006), through colourful particles of Interpretative repertoires in the kaleidoscope from the happy versus sad and then teaching versus learning spectrums. What is more, when observing inside the kaleidoscope, we come to see that some other sub-themes, such as a ‘sad senior’ versus ‘energetic junior’ spectrum, had been formulated in connection with the different levels of online teaching capabilities. In this way, the interlocutors implicitly formulated a commonplace of HE institutions in the era of the new normal: Technology-oriented facilitation becomes a key skill for future professors.

### Slogan as Commonplace in China

Two aspects of the ideological dilemmas seem evident. Firstly, what the slogan, ‘Suspending Classes without Stopping Learning’, advocates could be partially realised at an institutional level, but not fully. As the senior professor we interviewed told us, her university might be able to do so, but perhaps not all others could do so while still maintaining a high level of quality (lines 6–9 of Extract 3). Secondly, the realisation of this institutional ideology depends much on the particularity of the online teaching method. A feeling of uncertainty regarding the teaching–learning dilemma is common among many teachers. Another issue concerns what online teaching is able to do and, moreover, do it best; what it is incapable of doing; and what we believe is important for traditional face-to-face teaching to add. The ideological dilemmas among Chinese professors are not about requiring them to accept a difficult choice between online or onsite teaching, but how they place themselves appropriately depending on the disciplines and content/subject matter and the forms of knowledge. The interactions among Chinese professors suggest that future teaching and learning at the HE institutions, at least in China, might be developed into a blended style with distance and onsite learning.

### Dilemmas Over Teachers’ Blended Teaching in the UK

The case of the British HE teacher, T, can be seen, in a sense, to acknowledge and reproduce this account as an aspect of lived ideology or common sense understanding of online teaching and of hybrid approaches such as blended learning. T’s proposed solution to this ideological dilemma (‘the best of a bad job’, ‘inevitable’, ‘impossible’, and ‘not much of an option’ in lines 22–26, Extract 6) might not meet with the approval of others, but what none of them could deny is that such a dilemma exists during the pandemic. HE teachers are on a battleground upon which the struggle between these opposing ideals (e.g. online teaching as ‘not new’ to certain programmes as well as online teaching being ‘extended’ to normal face-to-face teaching programmes, blended teaching according to the university versus blended teaching according to HE teachers such as T) are played out. Moreover, the ways in which HE teachers conduct their day-to-day teaching practices seems to depend on how they position themselves within this ideological field during the pandemic.

## Conclusion

In this paper, we have shown that our discursive psychology-led analysis of the HE teacher participants’ interview talk made visible the ideologically dilemmatic nature of the participants’ views on online teaching that was necessitated and enforced due to the pandemic. In the Japanese examples of interview talk, the ideological dilemmas of online teaching are focused on generational difference. The Japanese teachers’ views are shaped by the local interactional context, in this case, a culturally pervasive hierarchy according to age and gender. Chinese and British HE teacher participants orientate to the importance of blended learning in the post-pandemic period. In particular, the teacher participants in the different settings formulate the significance of blended learning differently, orienting to the history of their own department and the expertise that they can make use of. This may suggest that communication between the university administration and teachers can enable a version of blended learning that is suitable to the local context of the programme and department.

Our analysis highlights the variability of ideological dilemmas (Billig, 1989). Various discursive actions, namely, commonplaces and Interpretative repertoires, are used by the teachers when they justify their positions and accommodate their views to the local contexts. HE teachers’ ideological dilemmas are related to the different levels of experience of implementing online teaching, as well as the different forms of assessments, knowledge types, and ways of conceptualising and implementing blended learning between the universities and HE teachers. We have discussed the notion of ideological dilemmas being related to the contrary themes of common sense, e.g. contrary maxims and opposition views. Various commonplaces have emerged in the context of the interaction. HE teachers have been seen to oscillate between different positions on the topic of online teaching. Oscillation identified in the analysis indicates the presence of ideological dilemmas, as the teachers switch back and forth between two or more equally balanced but contradictory aspects of the pandemic pedagogy’s common sense. ‘In many respects, common sense resembles a kaleidoscope. A limited number of elements is continually twisted into an infinite number of new configurations’ (Billig, 1992, p. 17). We have seen the teachers turning the kaleidoscope as they oscillate between positions. In facing the enforced online learning-only platform, they actively and positively accommodate their views to what they consider appropriate in the given context and to how they can deliver the teaching required. There are attempts to present their ‘mitigated’ positions, without exerting strong views (Billig, 1989). Therefore, our analysis contributes to a nuanced understanding of HE teachers’ views on their teaching practices during the pandemic, ultimately highlighting what is called ‘the social nature and content of thought’ (Billig et al., 1988, p. 2). We consider the variability between the HE teachers’ views and the ways they expressed them to be the hallmark of a fine-grain construction process of multiple HE teachers’ views towards enforced online teaching in those three HEI contexts (Table 1).

Our small-scale, exploratory study did not reveal national or cultural differences in the HE discourse systems (e.g. Scollon et al., 2012) as some may have expected. However, regardless of cultural differences in communication, online teaching has become an indispensable part of reality for today’s HE teachers, encompassing their teaching skills and capabilities, their institutions, their countries, their traditions and future, their students, and, most importantly, the significance of their role in HE institutions. Our analysis reveals a myriad of ideological dilemmas: Online teaching is not a replacement for onsite/face-to-face teaching. Rather than treating the pandemic’s impact on HE teaching as being merely a disruption for the teachers, our interview-based research afforded an opportunity for teachers to reflect on their teaching practice and to think about their fundamental role at HEIs and the various ways in which to accommodate their teaching to the new normal. Along with the aforementioned research on HE teachers’ views and perceptions of teaching during the pandemic, our research has shown that online teaching as emergency remote teaching will build on future research to transform the ways in which higher education will be delivered in future (Gallagher & Palmer, 2020).

## Appendices

**Table 1:** Conventions used for transcribing prosody (Atkinson & Heritage, 1984)

[]	Overlapping utterances
(.)	Micropause
=	Latched utterances
↑	Rising intontation
↓	Falling intonation
(0.8)	Measured pause (seconds)
house	Accentual emphasis
°they°	Quieter speech
[…]	Omitted speech
(there)	Doubtful transcription
((*coughs*))	Description of action
YE:EH	Capital letters: especially loud speech

**Table 2:** A list of participants and interview details for the analysis

University	Pseudonyms and Participant details	Gender	Extracts	Interview details	Via/duration
University J (Japan)	Y, associate professor, studied in the USA and Japan	Female	1 and 2	Twice (early and late November 2020) Interviewed by Kondo in English and Japanese	Zoom, 35 min
University C (China)	P01, senior academic, professor	Female	3	late December 2020 and January 2021, Interviewed by Hong in Chinese	Participant 01’s office, then online, 30 min
	P02, junior lecturer,	Female	4	late December 2020, Interviewed by Hong in Chinese	Hong’s office, then online, 30 min
	P03, senior academic, professor	Female	5	Interviewed by Hong in Chinese	Online, 26 min
	P04, junior lecturer	Female	Not included	Interviewed by Hong in Chinese	Online, 27 min
University B (UK)	T1, senior academic	Male	6 and 7	December 2020 Interviewed by Murakami in English	Zoom, 38 min
	T2, junior lecturer	Female	Not included	December 2020 Interviewed by Murakami in English	Zoom, 1.5 h

## Footnotes

1 We are aware of the conceptual issue of whether teaching and learning should be treated as separate or otherwise. Since it is not the main subject of this paper, we defer the discussion and use the term ‘teaching’ to refer to both teaching and learning.

2 In our work, we have come across multiple terms that are synonymous with, or refer to one of the several variants of, online teaching and learning: distance learning, remote teaching and learning, internet lecture, and distance education (see also Rapanta et al., 2020). Likewise, what is not online teaching is referred to as, for example, face-to-face teaching, classroom teaching, or on-site teaching.

3 For the sake of consistency of terms in this article, we use ‘online teaching’, which also includes ‘online learning’ or other sibling terms (see Footnotes 1 and 2).

4 Please see Table 2 in the appendices for details of the participants and interview activity details.

5 2 Japanese, 2 British, and 4 Chinese HE teachers.
